# Delirium Tremens

**Published:** 1874-02-01

**Authors:** N. S. Davis


### DELIRIUM TREMENS.—FATTY DEGENERATION.

### CLINICAL CASES IN THE MEDICAL WARDS OF MERCY HOSPITAL. SERVICE OF PROF. N. S. DAVIS.

WITH the table filled with a number of fresh morbid specimens, the lecturer addressed his class substantially as follows:

Delirium tremens, or that form of temporary mental derangement caused by the use of alcoholic drinks, is, unfortunately, of frequent occurrence in almost all populous communities; and the wards of our hospital are seldom entirely free from cases of this class. The subject from which these morbid specimens were taken was a man of intelligence, between twenty-five and thirty years of age, naturally strong, and well formed, but had accustomed himself to the use of alcoholic drinks for many years. It was stated by his friends that for three weeks before his admission to the hospital he had been almost constantly intoxicated, much of the time taking from one to two pints of brandy per day, while during the same time he took very little food. At the time of his admission, and for three or four days previous, he had been exhibiting all the phenemenon of *mania a potu*, or the delirium of the drunkard. When first seen in the ward, his face was pale, or rather of a purplish color; eyes sunken; the vessels of the conjunctiva distended with blood; and pupils large; the expression of countenance haggard; the extremities cool and blue; the pulse small, weak, and frequent; the stomach so irritable that almost everything swallowed was quickly rejected by vomiting, accompanied by a dark greenish fluid, mixed with mucus; and motions indicating great epigastric distress.

His mind was constantly occupied with all sorts of horrid images and phantoms; and he was much of the time engaged in a struggle to get out of bed and away from his attendants. There was constant vigilance, and much muscular agitation, or tremor. He could not be kept sufficiently quiet to permit a direct physical examination of the cardiac and hypochondriac regions; but the general symptoms justified a very unfavorable prognosis. And yet, up to that time, his friends continued to give him the alcoholic drinks.

Their further use was forbidden; the attendants were directed to use no more force in restraining him than was absolutely necessary to prevent him from doing injury to himself; and the following prescriptions were ordered:

℞.—Carbolic acid, cryst., 8 grs.
Glycerine, ℥ ss.
Tinct. digitalis, ℥ i.
Camph. tinct. opium, ℥ iijss.

Mix. Give one teaspoonful, in a tablespoonful of water, every two hours. It was hoped that this might allay the gastric irritation, steady and strengthen the heart's action, while the camphorated tincture of opium would lessen the morbid vigilance, without impairing the action of the kidneys, or endangering excessive narcotism. In the evening, the narcotic effect was to be increased by a single dose of fifteen grains each of bromide of potassium and hydrate of chloral. Tablespoonful doses of milk with lime-water, were also directed to be given, every two hours, alternately with his medicine.

On the following day, the condition of the patient was in no respect improved. The attendants had succeeded only very partially in carrying out the directions, the patient resisting the taking of either nourishment or medicine; while one of his friends had smuggled in a small bottle of what he called "good brandy," some of which had evidently been used. The matters vomited were becoming more dark and grumous; his pulse more feeble; and he died on the evening of the third day after admission. A *post-mortem* examination revealed no important morbid appearances visible to the unassisted eye, except in the stomach, duodenum, liver, and kidneys. These organs are before you, fresh as they were taken from the body. The stomach and duodenum are laid open; you see the mucous membrane, in its whole extent, presenting an intensely red and tumefied condition. In some places, where most intensely injected with blood, the surface is dark brown, and, apparently, softened. These appearances are the result of severe inflammation in the gastro-duodenal mucous membrane. And this inflammation was probably the direct cause of death.

The kidney is seen to be moderately enlarged; rather soft or flabby to the feel; and, on being laid open, the cortical, or secreting structure, is pale, and several small masses of fatty tissue at different points are observable. No analysis of the urine was made.

The liver is seen to be greatly enlarged, being more than twice its natural size. Its color is light olive, both internally and externally; and its increased bulk is plainly owing to infiltration, or deposit of fat globules, constituting the most common form of *fatty liver*. The heart is also loaded with fat; and its muscular tissue paler than natural. These morbid specimens fully illustrate the two leading effects of alcoholic drinks on the physical organization of the human body. The fatty degenerations in the liver, heart, kidneys, etc., are the result of the slow, long-continued, moderate influence of alcohol in retarding the oxydation of the carbonaceous matters of the system, and allowing it to accumulate in the form of inert fat; while the acute gastro-duodenitis is the result of the direct irritating influence of strong distilled spirits, taken in large quantities, without ordinary food.

Some have expressed doubts as to whether alchoholic drinks were capable of producing direct inflammation of the mucous membrane of the stomach; but such inflammation is certainly a frequent complication of delirium tremens, and adds greatly to the danger of that disease. It is very generally supposed that the delirium and trembling, result from the sudden withdrawal of the so-called, stimulating drink, and the consequent anæmic condition of the brain. And it is certainly true, that, in many cases, the first indications of delirium occur from one to five days after the inebriant has been discontinued. But it is equally certain that, in two-thirds of all the cases that have come under my observation, the symptoms supervened, while the patients were still in the full supply of their accustomed drink. Whenever the alcoholic beverage is kept in contact with the brain structures, constantly retarding the molecular changes for a considerable time, while the supply of nutritive matter through the digestive organs is suspended, or greatly deficient, that perversion of function which is styled delirium tremens ensues, whether the drink is continued or not. In simple, ordinary cases of delirium, not complicated with any serious disease in the chest or abdomen, the indications for treatment are simple, and easily fulfilled. The patient should be kept at rest, with kind, persuasive, encouraging words, and as little physical or forcible restraint as possible. All alcoholic drinks should be entirely discarded, and, in their place, such medicines given as will exert a soothing, tranquilizing influence, favoring sleep at night; and such bland nourishment as will be most readily retained and assimilated. From ten to fifteen grs. of bromide of potassium, given in solution with the same number of minims of the tincture of digitalis, every two or three hours, according to the degree of excitement, and from fifteen to twenty grains of hydrate of chloral, between eight and nine o'clock in the evening, will be all the medicine needed in most of these cases. Nourishment is of even more importance than medicine. At first, the patient should be induced to take two or three tablespoonfuls of milk, beef-tea, or other simple liquid nourishment, between each of the doses of his medicine; and after he begins to recover, the food may be more varied, and in larger quantities. In cases accompanied by paleness, constant sweating, a small weak pulse, and scanty urine, the following may be given between each of the doses of the bromide and digitalis:

R.—Carb. ammon., ℨ ij.

Camph. tinct. opium, ℥ ij.

Camph. water, ℥ iss.

Simple syrup, ℥ ss.

Give one teaspoonful every two or three hours, in a tablespoonful of water.

In cases accompanied by such persistent vomiting of thin mucus, of a green or brownish color, as indicates special gastro-duodenal inflammation, a powder containing one grain of calomel and one-quarter of a grain of sulphate of morphia, given every three hours, and tablespoonful doses of cold milk and lime-water, have often succeeded well in gaining control over both the delirium and gastric irritation. After the first day, the calomel should be omitted, and its place supplied by five grains of subnit. of bismuth, or three grains of oxide of zinc, with the same quantity of morphia as before. In a few instances, after the mental excitement and gastric irritability had much abated, a troublesome hiccough has supervened, which has yielded to five grain doses of monobromated camphor more readily than to any other remedy. There has been, and still is, a tendency to treat delirium tremens too heroically; that is, to give too large doses of medicine, either by the mouth or hypodermically.

We cannot but regard twenty, thirty, or forty grain doses of chloral, half grain, and grain doses of morphine, or fluid drachm doses of tincture of digitalis, as dangerous and unnecessary. I have never resorted to such doses; but several cases have come under my observation in which they have been resorted to, some of which terminated suddenly fatal. About two years since, a case was admitted into this hospital, in the early stage of delirium tremens. He was a middle-aged man, of good physical development; and one of the assistants in the hospital gave him, at once, about *fifty grains* of hydrate of chloral. It was followed, in a short time, by narcotism, so profound that artificial respiration had to be maintained for three hours before he regained a condition of safety. In a disease involving so much impairment of nutritive and molecular changes, a milder medication, and more attention to nourishment, is the safer course.

